# Characterization and Genetic Analysis of Rice Mutant *crr1* Exhibiting Compromised Non-host Resistance to *Puccinia striiformis* f. sp. *tritici* (*Pst*)

**DOI:** 10.3389/fpls.2016.01822

**Published:** 2016-11-30

**Authors:** Jing Zhao, Yuheng Yang, Donghe Yang, Yulin Cheng, Min Jiao, Gangming Zhan, Hongchang Zhang, Junyi Wang, Kai Zhou, Lili Huang, Zhensheng Kang

**Affiliations:** ^1^State Key Laboratory of Crop Stress Biology for Arid Areas, College of Plant Protection, Northwest A&F UniversityYangling, China; ^2^State Key Laboratory of Crop Stress Biology for Arid Areas, College of Life Science, Northwest A&F UniversityYangling, China; ^3^Shaanxi Rice Research Institute, Hanzhong Agricultural Science InstituteHanzhong, China

**Keywords:** non-host resistance, rice mutant, wheat stripe rust, defense-related genes, genetic mapping

## Abstract

Wheat stripe rust, caused by *Puccinia striiformis* f. sp. *tritici* (*Pst*), is one of the most devastating diseases of wheat in China. Rapid change to virulence following release of resistant cultivars necessitates ongoing discovery and exploitation of new resistance resources. Considerable effort has been directed at non-host resistance (NHR) which is believed to be durable. In the present study we identified rice mutant *crr1* (compromised resistance to rust 1) that exhibited compromised NHR to *Pst*. Compared with wild type rice variety Nipponbare, *crr1* mutant displayed a threefold increase in penetration rate by *Pst*, and enhanced hyphal growth. The pathogen also developed haustoria in *crr1* mesophyll cells, but failed to sporulate. The response to the adapted rice pathogen *Magnaporthe oryzae* was unchanged in *crr1* relative to the wild type. Several defense-related genes involved in the SA- and JA-mediated defense pathways response and in phytoalexin synthesis (such as *OsPR1a*, *OsLOX1*, and *OsCPS4*) were more rapidly and strongly induced in infected *crr1* leaves than in the wild type, suggesting that other layers of defense are still in effect. Genetic analysis and mapping located the mutant loci at a region between markers ID14 and RM25792, which cover about 290 kb genome sequence on chromosome 10. Further fine mapping and cloning of the locus should provide further insights into NHR to rust fungi in rice, and may reveal new strategies for improving rust resistance in wheat.

## Introduction

Wheat stripe rust, caused by *Puccinia striiformis* f. sp. *tritici* (*Pst*), is a devastating disease of wheat worldwide (Wellings, 2011). In China, the annual yield losses to stripe rust in wheat were estimated to be about 1 million metric tons (Chen et al., 2009). Cultivation of resistant varieties is the most effective, economical, and environmentally friendly way to control the disease. Although many resistance genes have been identified and utilized in wheat cultivars (Cheng et al., 2014; Lu et al., 2014; Han et al., 2015), the protection conferred has not been durable due to genetic variation in the pathogen population. One example is that of resistance gene *Yr26* that was widely used in Chinese wheat breeding in recent years. New *Yr26*-virulent (CYR34, V26) races are now increasing and are causing unacceptable levels of disease on many of the cultivars with *Yr26* (Han et al., 2015). Although resistances governed by quantitative trait loci (QTL) confer a broader-spectrum resistance, the level of resistance is not adequate to prevent significant crop losses, especially under severe epidemic conditions (Niks et al., 2015b). Thus, more durable control of stripe rust is urgently needed, and in addition to current exploration and identification of new resistance genes in wheat and its close relatives, a better understanding of non-host resistance (NHR) may offer opportunities in breeding for sustainable disease control.

In nature, a specific pathogen usually causes disease on a few plant species; that is, most plants are resistant to a wide range of phytopathogens. This form of disease resistance exhibited by all members of a plant species to all genetic variants of a non-adapted pathogen species [or possibly *formae speciales* (f. sp.)] is known as NHR. Due to its broad-spectrum effectiveness and durability NHR is of considerable interest for crop resistance improvement.

The genetic and molecular mechanisms underlying NHR remain largely unknown. Currently, the best-studied example of NHR is interaction between Arabidopsis and the non-adapted barley biotrophic fungal pathogen *Blumeria graminis* f. sp. *hordei* (*Bgh*), the causal agent of barley powdery mildew. Three NHR genes *PEN1*, *PEN2*, and *PEN3* required for penetration resistance of *Arabidopsis* to *Bgh* have been isolated. Functional mutants of any one of the three *PEN* genes display increased penetration rates by *Bgh. PEN1* (Collins et al., 2003; Lipka et al., 2005; Stein et al., 2006) encodes a membrane-associated syntaxin containing a SNARE (soluble N-ethylmaleimide-sensitive factor attachment protein receptor) domain and is a member of a large family of proteins functioning in membrane fusion and secretion events. Cytological studies have demonstrated that *PEN1* is involved in papilla formation. *PEN2* encodes a glycoside hydrolase, which associates with the periphery of peroxisomes. *PEN3* encode an ABC (ATP binding cassette) transporter that is localized to the plasma membrane. PEN2 and PEN3 may collaborate in transport of antimicrobial compounds to the apoplast. Importantly, the protein products of these *PEN* genes accumulate at sites of fungal penetration. Moreover, these genes also contribute to *R* gene-mediated resistance and cell death in response to both adapted and non-adapted pathogens (Johansson et al., 2014). While, for those plants that are evolutionary closely related to the natural host, the NHR is proposed to be predominantly mediated by multiple R genes that collectively confer resistance to all isolates of a pathogen species (Schulze-Lefert and Panstruga, 2011). For example, the NHR of Arabidopsis to non-adapted *Albugo candida* may result from recognition of pathogen effectors by multiple WRR4-like genes, which encode typical NB-LRR resistance proteins (Borhan et al., 2008).

There are many published reports on NHR to rust fungi. For example, NHR of Arabidopsis and Medicago to bean rust (Patto and Rubiales, 2014; Ishiga et al., 2015; Langenbach et al., 2016), barley and *Brachypodium distachyon* NHR to cereal rust pathogens (Zellerhoff et al., 2010; Dawson et al., 2015; Figueroa et al., 2015; Niks et al., 2015a). As the model of monocotyledonous plant, rice is unusual in not being affected by a rust pathogen (Ayliffe et al., 2011b). Several studies indicated that rust fungi have some potential to infect rice and trigger host defense responses such as production of reactive oxygen species and accumulation of Pathogenesis-related proteins (Ayliffe et al., 2011a; Li et al., 2012). Thus, it is of interest to characterize non-host interaction between rice and cereal rust pathogens and identify key genes involved in NHR. Proteomic studies revealed proteins that are involved in phytoalexin production, and glycerol-3-phosphate metabolism may have a role in rice NHR to *P. triticina* and *Pst* (Li et al., 2012; Zhao et al., 2014).

In the present study we identified rice mutant *crr1* (compromised resistance to rust 1) that allowed a high level of penetration rates by *Pst* and enhanced hyphal growth. The fungus was able to develop haustoria in mesophyll cells of the mutant, but failed to sporulate. Histological analysis revealed that hydrogen peroxide (H_2_O_2_) production and callose deposition were not affected in the *crr1* mutant. Furthermore, upon infected by *Pst crr1* showed strikingly enhanced expression levels of defense-related genes involved in the SA-, and JA-mediated defense pathways as well as phytoalexin synthesis. These observations suggested different molecular mechanisms underlying NHR to *Pst* in rice compared to the host resistance. Genetic analysis demonstrated that the phenotype of *crr1* was conditioned by a recessive gene between markers ID14 and RM25792 at the end of rice chromosome 10. Characterization and genetic study of *crr1* would provide new insights into NHR, and assist in breeding wheat cultivars with durable resistance to stripe rust.

## Results

### *crr1* Exhibited Compromised NHR to *Pst*

**FIGURE 1:**
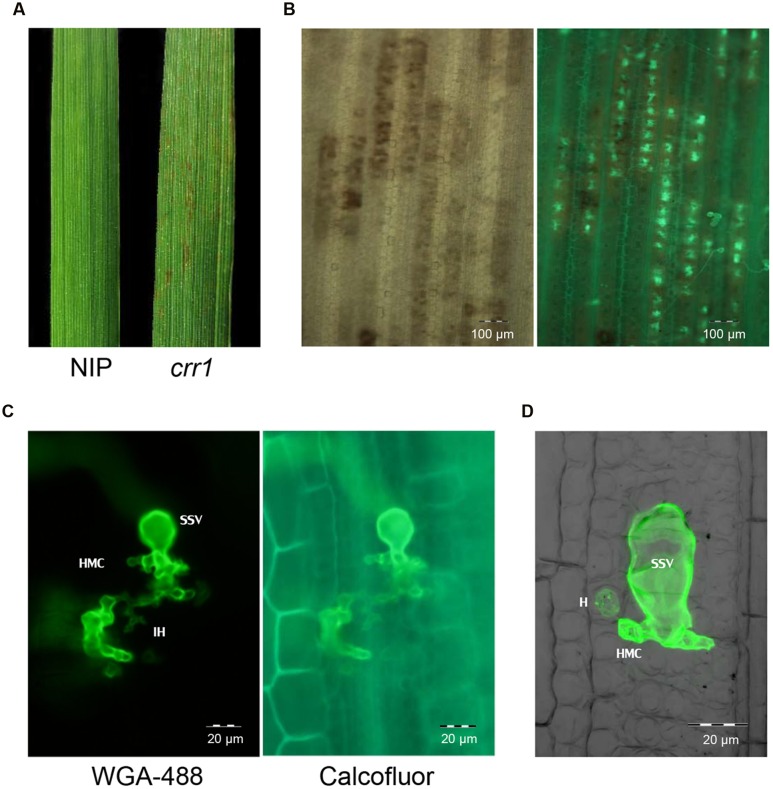
**Infection of rice mutant *crr1* and wild type Nipponbare with *Puccinia Striiformis* f.sp. *tritici* (*Pst*) race CYR32.** **(A)** Macroscopic observation of Nipponbare and *crr1* leaves infected with *Pst*. **(B)** Microscopic observation of *Pst* development in *crr1* leaves using bright field (left) and fluorescence (right). **(C)** *Pst* infection site in *crr1* showing formation of sub-stomatal vesicle (ssv), infection hyphae (IH) and haustoria mother cell (HMC). **(D)** A haustorium (H) in *crr1* mesophyll cells. The image was composite of stacked fluorescent and blight field photographs by confocal microscopy.

**FIGURE 2:**
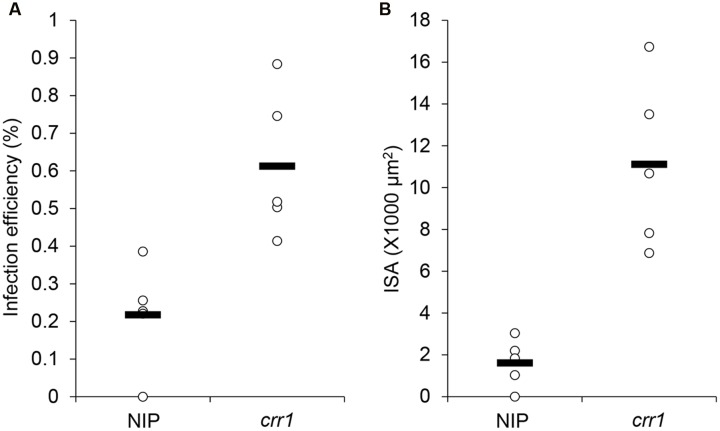
**Penetration frequency and infection site area of *Pst* on Nipponbare and *crr1*.** **(A)** Penetration frequency of *Pst* on Nipponbare and *crr1*. **(B)** Infection site area of *Pst* on Nipponbare and *crr1*. Open circles show measurements for each plants. Black lines show the medians of the data.

We screened 5,229 T2 rice mutant families and found nine putative mutants that allowed increased *Pst* growth in leaf tissue. These putative mutants were designated as Comprised resistance to rust fungus (*crr1*-9). Among them, *crr1* showed the most *Pst* development in rice tissues. At 14 days after inoculation with *Pst*, wild type plants showed no visible symptoms, whereas brown flecks appeared on leaves of *crr1* mutant plants, and these lesions were associated with hyphal colonization (**Figures 1A,B**). Microscopic observation revealed that most of the urediniospores germinated on the leaf surfaces of both wild type and *crr1*. However, on the wild type rice, only a few (0.2%) germinated urediniospores penetrated into stomates and successfully formed substomatal vesicles (ssv). In contrast, on *crr1*, the penetration rate was 0.6%, about three times higher than that on wild type plants (**Figure 2A**). Moreover, the rust fungus can produce haustoria in *crr1* mesophyll cells, suggesting a defective of pre-invasion NHR in the mutant (**Figures 1C,D**). In addition, compared with the small colony (from 0 to 4,000 μm^2^ in area) developed in wild type rice, the clearly larger colonies were developed in *crr1* with an average area over 10,000 μm^2^ (**Figure 2B**).

To determine whether disease response of *crr1* was affected following infection by an adapted pathogen, we compared the responses of mutant and wild type plants to *Pyricularia oryzae* (strain Guy11), the causal agent of rice blast. As shown in Supplementary Figure S1, both Nipponbare and *crr1* exhibited similar partial resistance responses to strain Guy11. These results suggesting that the host resistance is not affected by the mutation event in *crr1*.

### Transmission Electronic Microscopy Observation of *Pst* Growth in *crr1*

**FIGURE 3:**
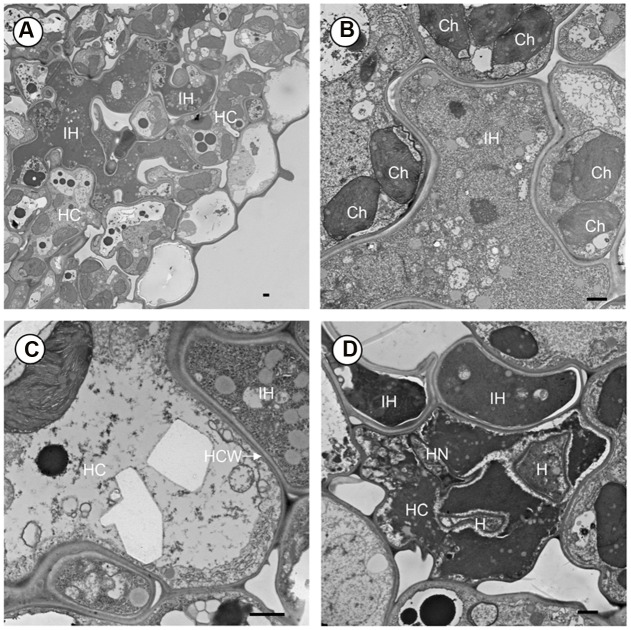
**Transmission electron microscope (TEM) observation of *Pst* development in leaf tissue of rice mutant *crr1*.** **(A)** Intercellular hyphae (IH) growing in the intercellular space and in the developing cavity between mesophyll and epidermis. **(B)** Intercellular hyphae (IH) surrounded by mesophyll cell. **(C)** Interface of host and fungus cell wall. **(D)** A haustorium in host cell and causing cell death. HC, host cell; IH, intercellular hyphae; Ch, chloroplast; HCW, host cell wall; H, haustorium; HN, haustoria neck. Scale bar: 1 μm.

To further understand the proliferation of *Pst* in *crr1*, we observed the colonization of *Pst* on *crr1* leaves at 14 dpi using transmission electronic microscopy. Extensive growth was evident in *crr1* leaf tissue (**Figure 3A**). More interesting, although at very low frequency, haustoria were observed in mesophyll cells (**Figure 3D**). Most of the haustoria were abnormal and were associated with host cell death. Host cells surrounding the hyphae remained living (**Figure 3B**) and there was no apparent cell wall thickening or papillae formation (**Figure 3C**). Some hyphae had begun to die and there was no evidence of sporulation (**Figure 3D**).

### H_2_O_2_ Production and Callose Deposition in *crr1* Challenged by *Pst*

**FIGURE 4:**
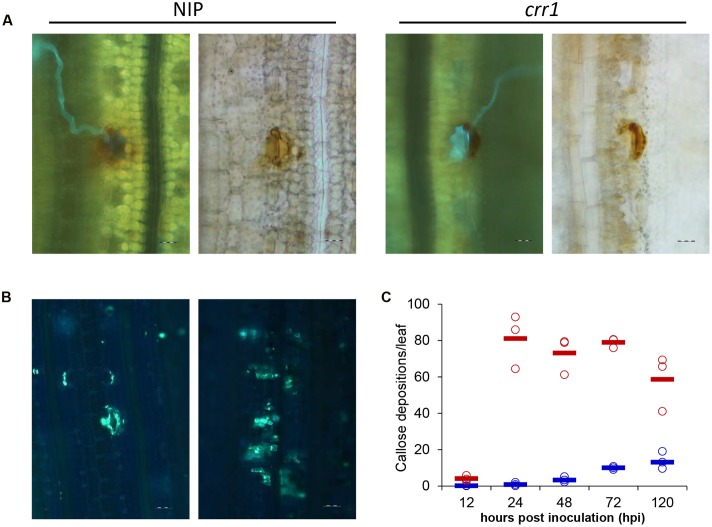
**Histochemical examination of hydrogen peroxidase production and callose deposition at *Pst* infection sites in Nipponbare and *crr1*.** **(A)** Fluorescence (left) and brightfield (right) images presenting hydrogen peroxidase production at infection sites in *crr1* and wild type plants 48 hpi by *Pst*, Bar = 20 μm. **(B)** Callose deposition in *crr1* and wild type plants at 120 hpi by *Pst*, Bar = 20 μm. **(C)** Quantification of callose deposition in *crr1* and wild type plants at 12, 24, 48, 72, and 120 hpi by *Pst*. Open circles show measurements for each plants. Solid lines show the medians of the data. Blue and red symbols stand for data from Nipponbare and crr1, respectively.

Hydrogen peroxide production plays a critical role in plant defense response. Previous studies have shown that H_2_O_2_ was induced in rice following rust infection (Ayliffe et al., 2011a; Yang et al., 2014). To examine whether the production of H_2_O_2_ was defective in *crr1*, we examined the H_2_O_2_ production at infection sites in mutant and wild type plants challenged by *Pst*. There was no apparent difference in H_2_O_2_ production between *crr1* and wild type plants (**Figure 4A**). H_2_O_2_ was produced in the guard cells at 24 hpi in both *crr1* and wild type plants, although some weak H_2_O_2_ signals were detected around the stomata. It is noteworthy that primary hyphae and ssv were formed in *crr1* at 48 hpi despite of the production of H_2_O_2_ around the infection sites. We postulated that H_2_O_2_ mainly functions as a signal molecule in rice-*Pst* interaction and the weakened resistance of *crr1* may result from lacking of some other downstream components. As another marker for plant defense response, callose deposition, was also compared between *crr1* and wild type plants using the aniline blue staining method. More extensive callose deposition was observed with the growth of pathogen in *crr1* (**Figures 4B,C**). These histological results suggested that the subdued NHR in *crr1* should be independent of H_2_O_2_ production and callose deposition.

### Relative Expression Levels of Defense Related Genes

**FIGURE 5:**
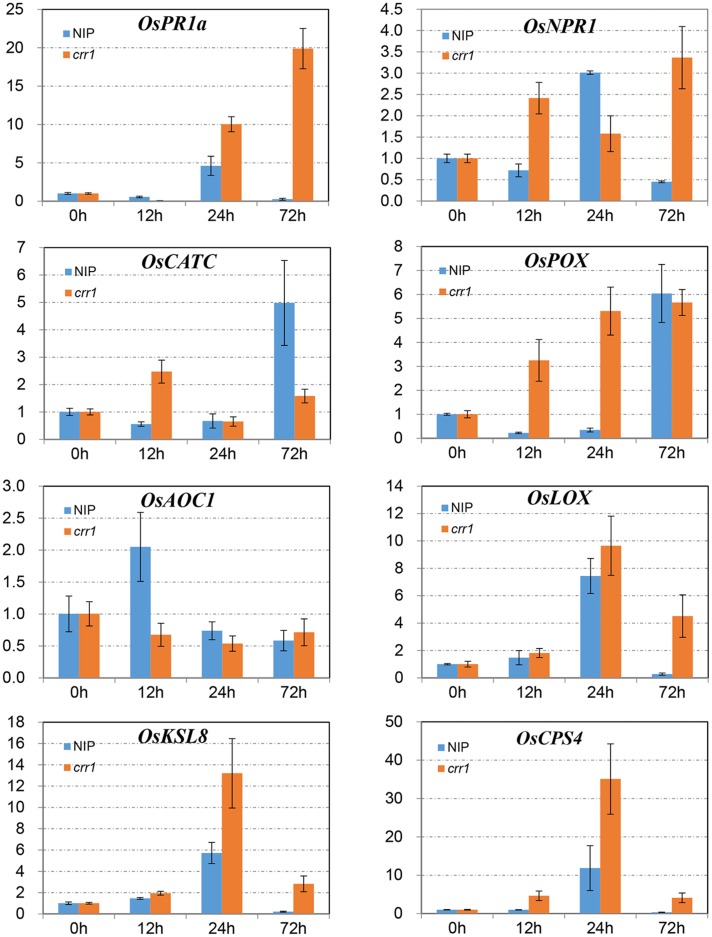
**Relative expression patterns of defense-related genes in Nipponbare and *crr1* infected with *Pst*.** The mRNA levels of eight defense-related genes in Nipponbare and *crr1* were examined at four time points (12, 24, 48, and 120 hpi). Relative expression levels are normalized to the values of the mock-inoculated plants.

In plant–pathogen interactions many defense-related genes have been identified, and their analysis in *crr1* could help in understanding factors involved in the NHR to *Pst*. Eight genes related to four functional categories were chosen to examine expression profiles. *NPR1* and *PR1* are marker genes for the salicylic acid-mediated defense pathway (Yuan et al., 2007; Yang et al., 2013). *OsCATC* and *OsPOX* encode catalase and peroxidase which function in ROS scavenging (Quan et al., 2008). *OsAOC1* and *OsLOX1* belong to the jasmonic acid pathway (Lyons et al., 2013; Yang et al., 2013). *OsCPS4* and *OsKSL8* represent genes involved in phytoalexin synthesis in rice (Hasegawa et al., 2010). Expression profiles of these genes were examined at four time points (0, 12, 24, and 72 hpi) in the mutant and wild type inoculated with *Pst* (**Figure 5**). The mRNA levels of *NPR1* were increased at 12 and 24 hpi in Nipponbare and *crr1*, respectively, and then declined in Nipponbare but remained at a high level in *crr1. PR1a* was induced at 24 hpi in both Nipponbare and *crr1. OsLOX1* was also induced at 24 hpi, while another gene *OsAOC1* involved in the jasmonic acid pathway was slightly induced at 12 hpi only in Nipponbare. Both *OsCPS4* and *OsKSL8* were induced at 24 hpi in Nipponbare and *crr1. OsCATC* was induced in *crr1* at 12 hpi, and at a considerably later stage (72 hpi) in Nipponbare. *OsPOX* was also induced at 12 hpi in *crr1* where it kept increasing. In general, most of the defense-related genes were responsive to *Pst* infection in both wild type and *crr1* mutant plants, but their induction was quicker and stronger in *crr1* compared with that in wild type plants.

### Mapping of the *Crr1* Gene

Sixty-six *F*_2_ individuals from the cross between *crr1* and Nipponbare were evaluated for response to *Pst* infection. Fifty-two plants exhibited compromised resistance similar to *crr1* and 14 plants displayed complete immune similar to Nipponbare. The phenotypic segregation fitted well to the expected 1:3 ratio indicative of a single gene model with homozygosity of the recessive allele leading to increased hyphal development at infection sites (χ^2^ = 0.81, *P* = 0.48). This observation suggested that the phenotype of *crr1* resulted from a single locus mutation. Southern-blotting results indicated that *crr1* possessed a single T-DNA insertion. The flanking sequence of the T-DNA was isolated by inverse PCR (Zhang et al., 2007) and the insertion site was located at the CDS of Os07g40020 (a GRAS family protein) but cosegregation analysis showed that the T-DNA insertion was independent of the locus segregating for the altered host response.

**FIGURE 6:**
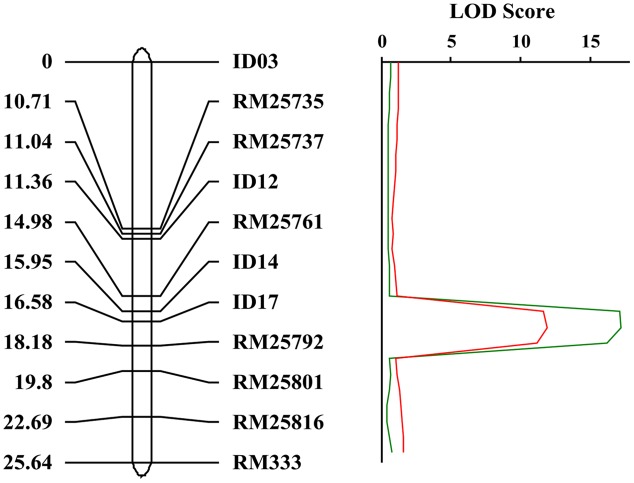
**Genetic map of the *crr1* locus based on the *crr1*/Zhonghua 11 F2 mapping population.** The candidate *crr1* gene was mapped to the region between two markers ID14 and RM25792 which located at the end of long arm of rice chromosome 10.

In order to map the gene, we developed *F*_2_ populations from crosses of *crr1* with Zhonghua 11 and Mudanjiang 8. Using bulked segregant analysis (BSA) (Michelmore et al., 1991) the *Crr1* locus was putatively mapped to the long arm of chromosome 10 and was linked to SSR marker RM25761. Twelve SSR and indel markers from this region were developed to generate a higher density map of the candidate region (**Supplementary Table S1**). Composite interval mapping (CIM) placed *Crr1* between markers Id14 and RM25792 (**Figure 6**) with a logarithm of odds (LOD) score of 11.6. Variation of infection site area (ISA) and NIS at the locus accounted for 29.8% and 39.9% of the total variation, respectively (**Table 1**). This candidate region encompasses about 290 kb of genome sequence and contained 40 annotated genes.

**Table 1:** Locus identified from composite interval mapping based on the infection site area (ISA) and number of infection sites (NIS) of the *crr1*/Zhonghua 11 F2 population inoculated with *Pst*.

Trait	Marker interval	LOD	PVE(%)^a^	Add^b^	Dom^c^
ISA	ID17-RM25792	11.9	29.8	4155.1	1773.9
NIS	ID17-RM25792	17.3	39.9	49.0	12.5


*^a^PVE, percentage of variation explained. ^b^Add, additive effect of allele. ^c^Dom, dominant effect of allele.*

## Discussion

Non-host resistance has potential to provide non-specific, durable resistance to diseases, and to offer alternative possibilities to traditional *R* gene-mediated resistance. Unfortunately, no rice gene underlying NHR to rust has been identified so far. In this study, we characterized and identified a gene conferring NHR to *Pst* using a rice mutant *crr1*. This study may contribute to our understanding of rice NHR to *Pst* and exploiting rice NHR to develop wheat lines with more durable resistance to *Pst*.

According to the two-layered paradigm of plant active immune system, there are two types of NHR: pre-invasion defense mediated by PAMP-triggered immunity (PTI) and post-invasion defense mediated by effectors-triggered immunity (ETI) (Gill et al., 2015). Our data showed that the frequency of stomatal penetration of *Pst* urediniospores on wild type rice (0.2%) was significantly reduced than that on wheat (over 25% in previous study) (Cheng et al., 2013). Similar studies demonstrated that 0.5% urediniospores of *P. triticina* formed ssv on *Arabidopsis* leaves (Shafiei et al., 2007). These results suggested that rice NHR to *Pst* might largely act at the pre-invasion stage. Histologic studies revealed H_2_O_2_ production and callose accumulation concentrated in and around guard cells following *Pst* inoculation, but no hypersensitive cell death was detected. Therefore, PTI mediated defense responses that precede host mesophyll cell invasion appear to play the predominant roles in blocking attempted *Pst*. Compared with intermediate hosts of *Pst*, such as barley and *Brachypodium*, rice is more distantly related to wheat and resistant to all rusts (Vogel et al., 2010; Mayer et al., 2011). Thus our results fit well with the molecular evolution model that PTI play a key role when pathogens attempt to infect more distantly related non-host species (Schulze-Lefert and Panstruga, 2011). Increased frequency of stomatal penetration and larger infection sites in *crr1* may result from a disrupted defense step preceding mesophyll cell invasion.

Although *crr1* plants exhibited more susceptibility to *Pst*, the H_2_O_2_ production was not affected relative to wild type. In addition, callose deposition increased as hyphal growth expanded in leaf tissues of the *crr1* mutant. Similarly, the induction of defense related genes, such as genes involved in SA/JA mediated defense pathway and phytoalexin synthesis, were quicker and stronger in *crr1* than that in wild type plants. Collectively, these findings suggest that increased development of *Pst* in apoplasts of *crr1* plants triggers a more extensive defense response. Counter-intuitively, these defense responses seemed to have limited effects on preventing the growth of *Pst*. Similar findings were reported for non-host interaction between *pen* mutants and the barley mildew pathogen (Collins et al., 2003; Lipka et al., 2005; Stein et al., 2006). A natural variant of *Arabidopsis* accession Wa-1 which has compromised resistance to non-adapted wheat leaf rust pathogen exhibits increased SA and PR1 expression following *P. triticina* challenge (Shafiei et al., 2007). In these cases, there seems to be a “true” NHR which spanning multiple layers of defense. *Crr1* mutants are indeed compromised for part of the defense (mainly related to ingress). However, the penetration rate is still very low and most defense responses still seem to be intact and in fact the pathogen cannot really proliferate/sporulate either.

Haustoria are special structures of biotrophic fungus that suppress host immune system and take up nutrients from host cells. Formation of haustoria is usually regarded as the symbol of parasitism establishment (Yi and Valent, 2013). Haustoria have been observed by fluorescence microscopy in several studies concerning rice-rust fungus interactions, while the details of haustoria is not clear yet (Ayliffe et al., 2011a; Yang et al., 2014). In the present study, we observed haustoria formation in mesophyll cells in rice mutant by TEM. This finding suggested that rust fungi have the potential to absorb nutrients and infect rice. However, only a limited number of haustoria were observed. Although urediniospores have stored nutrients for germination and initial infection, it was unbelievable that such a few hautoria can support the considerable colonization that encompassed hundreds of host cells in *crr1*. This suggests the possibility that hyphae are able to take up nutrients from the intercellular spaces. Although it is generally believed that the apoplast of plants leaves is a relatively nutrient-poor environment, a considerable number of microbes do derive nutrients from it. In a study of interaction between barley and *P. hordei*, sucrose and glucose were found in apoplast at much lower concentrations in infected than in healthy leaves, and uptake of hexoses by intercellular hyphae was suggested as the cause of the reduction (Tetlow and Farrar, 1993). A sucrose transporter SRT1 from *Ustilago maydis* was shown to take up sucrose from intercellular spaces of maize leaves allowing the hyphae to grow along the phloem (Wahl et al., 2010). Thus, we postulated that hyphae of *Pst* may obtain nutrients from the apoplast in leaves of the *crr1* rice mutant. These nutrients absorbed from the intercellular spaces support limited hyphal growth, but they may be insufficient to support sporulation.

Using BSA and CIM strategies we located the *crr1* locus between id14 and RM25792 at the end of chromosome 10. This region contains about 40 annotated genes, and there is no previously annotated host or non-host resistance gene in the interval. Recently, three genes involved in *Brachypodium* NHR to *Pst* were identified by flanking sequence isolation from mutant (An et al., 2016). Among them, *Bradi5g17540* encodes a BAP28 domain containing protein, *Bradi5g17540* encodes a MYB transcription factor and *Bradi5g11590* encodes a lipoxygenase. However, none of them is present in the candidate region of *crr1*. Another gene conferring non-host resistance to *P. striiformis* was identified in barley, and designated as *Rps6* (Andrew et al., 2016; Li et al., 2016). *Rps6* was mapped on the long arm of barley chromosome 7H, which has no collinearity with rice chromosome10, suggesting that it is different with *crr1* locus.

Several studies have demonstrated the feasibility of transferring single NHR-related genes across plant species to create durable, broad-spectrum resistance (Brutus and He, 2010; Wen et al., 2011; Schoonbeek et al., 2015; Langenbach et al., 2016). The mechanisms of rice resistance to *Pst* can be used to improve wheat resistance. Since it is not feasible to transfer gene through homoeologous recombination by hybridization, transgenic strategy might be a good alternative. Thus, further studies based on more comprehensive efforts will be needed to clone the corresponding rice gene conferring NHR to stripe rust fungus.

## Materials and Methods

### Plants and Growth Conditions

Rice mutant *crr1* was identified from 5229 Nipponbare mutants made by T-DNA insertional mutagenesis at Huazhong Agricultural University, Wuhan, China (Wu et al., 2003). Two segregating F2 populations derived from crosses of *crr1* with two highly resistant japonica varieties Mudanjiang 8 (MDJ8) and Zhonghua11 (ZH11) identified previously (Yang et al., 2014).

### Maintenance and Inoculation of *Pst*

*Pst* isolate CYR32 (a predominant *Pst* race in China) were maintained on a susceptible wheat cultivar, Mingxian 169, following the procedures and conditions described by Zhang et al. (2011). For inoculation of rice, 3-week-old rice seedlings were pre-spayed with 0.1% Triton X-100, then fresh *Pst* urediniospores suspensions (50 mg urediniospores ml^-1^) were applied with a fine paintbrush onto the adaxial surface of the second leaf. Rice plants inoculated with sterilized distilled water were used as a negative control. The inoculated seedlings were kept in a dew chamber at 100% humidity for 36 h at 12°C in complete darkness to ensure the maximal rate of infection. Subsequently, the seedlings were transferred to a growth chamber at 16°C with a 16/8 h light/dark cycle. Leaf tissues were collected at specific time points for various analyses.

### Histochemical Analysis of the Rice-*Pst* Interactions

At least six inoculated rice leaves were harvested at 10 days post inoculation (dpi) for histopathological analysis. Rice leaf segments of 4 cm were cut from the center of inoculated leaves. Leaf sections were fixed and decolorized in ethanol/trichloromethane (4:1, v/v) containing 0.15% (w/v) trichloroacetic acid for 2 days, and the fixation solution was replaced with fresh solution twice every other day. The specimens were cleared in saturated chloral hydrate until leaf tissues were translucent. For Calcofluor White (Sigma–Aldrich) staining, the method described by Zhang et al. (2011) was followed. For further visualization of internal infection structures, the wheat germ agglutinin (WGA) conjugated to the Fluorophore Alexa 488 (Invitrogen) staining was used (Ayliffe et al., 2011b). H_2_O_2_ was detected using the 3, 3-diaminobenzidine (DAB, Amresco, Solon, OH, USA) staining method (Thordal-Christensen et al., 1997) and observed under differential interference contrast (DIC) optics.

### Data Collection and Analysis

For ISA and penetration frequency, rice leaves were harvested at 2 weeks post inoculation (dpi) and stained by Calcofluor for observation. Infection sites were photographed under 10× or 20× magnifications using a focal plane that maximized the area of each infection site. ISA was measured using Olympus CellSens^®^ Digital Imaging Software (Version 1.5). At least 10 infection sites were measured for each individual F2 rice plant. Penetration frequencies were calculated using the number of infection sites developing ssv or hyphe divided by the number of all urediospores and 10 inoculated plants of Nipponbare and *crr1* were scored per experiment.

For the quantification of callose deposition by *Pst* infection, rice leaves were harvested at 12, 24, 48, 72, and 120 hpi. A total of 30 infection sites were examined for each time point of Nipponbare or *crr1* plants in one experiment and three independent experiments were performed.

For all data, means and standard errors were calculated from three independent biological replicates using Student’s *t*-tests.

### Cytological Analysis of the Rice-*Pst* Interaction

Leaves were harvested from inoculated rice *crr1* mutants at 2-week after inoculation and prepared for transmission electron microscope (TEM) examination according to procedures previously described (Zhang et al., 2011). The leaf samples were cut into small pieces and fixed with 3% (v/v) glutaraldehyde in 50 mmol/l phosphate buffer (pH 6.8) for 3–6 h at 4°C. After rinsing thoroughly with the same buffer and post-fixation with 1% (w/v) osmium tetroxide for 2 h at 4°C, the samples were dehydrated in a graded alcohol series, embedded in gelatin capsules filled with LR White resin (Sigma–Aldrich), and polymerized at 60°C for 48 h. For TEM observations, ultra-thin sections of the samples were cut with a diamond knife and collected on 200 mesh copper grids. After contrasting with uranyl acetate and lead citrate, the grids were examined with a JEM-1230 TEM (Jeol Co. Ltd, Tokyo, Japan) at 80 kV.

### Genetic Mapping

Sequences of primers for SSR markers were obtained from The IRGSP (International Rice Genome Sequencing Project) (Matsumoto et al., 2005). For indel markers, polymorphisms markers primers were designed according to the DNA Polymorphisms information on NODAI Genome Research Centre^1^ (Arai-Kichise et al., 2011). The sequences of molecular markers were presented as **Supplementary Table S1**.

For bulk segregant analysis (BSA), genomic DNA was extracted from leaves of the parents and 163 F2 plants of *crr1*/Zhonghua 11 and 86 F2 plants of *crr1*/Mudanjiang 8. According to the data of the number of infection sites and ISA, five most susceptible and resistant F2 plants were selected and equal amounts of their DNA were mixed to form the susceptible bulk (SB) resistant bulk (RB). Genotypes for 96 polymorphism markers evenly distributed in rice genome were analyzed among SB, RB together with the parents. Genotypes from the parents and the bulks were used to identify molecular markers linked to the target loci.

Quantitative trait loci mapping for *crr1* locus was conducted based on the number of infection sites and ISA of each F2 plants. The inclusive composite interval mapping (ICIM) analysis was performed using the software QTL IciMapping V3.3. In the first step of the stepwise regression of ICIM, probabilities for including and excluding marker variables were set at 0.01 and 0.02, respectively. In the second step of interval mapping of ICIM, the threshold LOD score was set at 2.5 to declare significant QTL for all phenotyping methods.

## Author Contributions

ZK and JZ conceived and designed research. JZ and YY performed the genetic mapping and analyzed the data. JZ and DY performed the histological observation and gene expression analysis. GZ, HZ, and YC screen and identified the mutant. JW and KZ developed genetic population and molecular marker. MJ performed the TEM experiment. LH and ZK contribute comments during manuscript preparation. JZ wrote the manuscript and ZK revised the manuscript. All authors read and approved the final manuscript.

## Conflict of Interest Statement

The authors declare that the research was conducted in the absence of any commercial or financial relationships that could be construed as a potential conflict of interest.

## Footnotes

1 http://www.nodai-genome.org/oryza_sativa_en.html

## Supplementary Material

**TABLE S1 | Primer sequences of molecular markers for *crr1* genetic mapping.**
