# Supplementary material for: Characterization and Genetic Analysis of Rice Mutant crr1 Exhibiting Compromised Non-host Resistance to Puccinia striiformis f. sp. tritici (Pst)
Source: Front Plant Sci. 2016 Nov 30;7:1822. doi: 10.3389/fpls.2016.01822 (PMC5127839; doi:10.3389/fpls.2016.01822)
Supplement: Supplementary file 2 [file Image_1.PDF]

Supplementary figure 1

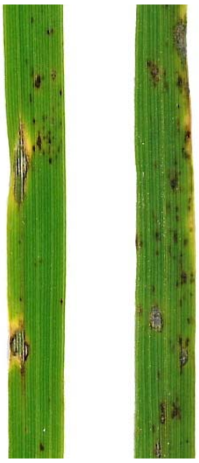

Figure S1, Leaves from Nipponbare (NIP) and *crr1* inoculated by *Pyricularia oryzae* (strain Guy11).
